# Characterising non-urgent users of the emergency department (ED): A retrospective analysis of routine ED data

**DOI:** 10.1371/journal.pone.0192855

**Published:** 2018-02-23

**Authors:** Colin O’Keeffe, Suzanne Mason, Richard Jacques, Jon Nicholl

**Affiliations:** Centre for Urgent and Emergency Care (CURE), School of Health and Related Research (ScHARR), University of Sheffield, Sheffield, United Kingdom; Klinikum rechts der Isar der Technischen Universitat Munchen, GERMANY

## Abstract

**Background:**

The pressures of patient demand on emergency departments (EDs) continue to be reported worldwide, with an associated negative impact on ED crowding and waiting times. It has also been reported that a proportion of attendances to EDs in different international systems could be managed in settings such as primary care. This study used routine ED data to define, measure and profile non-urgent ED attendances that were suitable for management in alternative, non-emergency settings.

**Methods:**

We undertook a retrospective analysis of three years of Hospital Episode and Statistics Accident Emergency (HES A&E) data for one large region in England, United Kingdom (April 1^st^ 2011 to March 31^st^ 2014). Data was collected on all adult (>16 years) ED attendances from each of the 19 EDs in the region. A validated process based definition of non-urgent attendance was refined for this study and applied to the data. Using summary statistics non-urgent attenders were examined by variables hypothesised to influence them as follows: age at arrival, time of day and day of week and mode of arrival. Odds ratios were calculated to compare non-urgent attenders between groups.

**Results:**

There were 3,667,601 first time attendances to EDs, of which 554,564 were defined as non-urgent (15.1%). Non-urgent attendances were significantly more likely to present out of hours than in hours (OR = 1.19, 95% CI: 1.18 to 1.20, P<0.001). The odds of a non-urgent attendance were significantly higher for younger patients (aged 16–44) compared to those aged 45–64 (odds ratio: 1.42, 95% CI: 1.41 to 1.43, P<0.001) and the over 65’s (odds ratio: 3.81, 95% CI: 3.78 to 3.85, P<0.001). Younger patients were significantly more likely to attend non-urgently out of hours compared to the 45–64’s (OR = 1.24, 95% CI: 1.22 to 1.25, P<0.001) and the 65+’s (OR = 1.38, 95% CI: 1.35 to 1.40, P<0.001). 110,605/554,564 (19.9%) of the non-urgent attendances arrived by ambulance, increasing significantly out of hours versus in hours (OR = 2.12, 95% CI: 2.09 to 2.15, P<0.001).

**Conclusions:**

Younger adults are significantly more likely as older counterparts to use the ED to obtain healthcare that could be provided in a less urgent setting and also more likely to do this out of hours. Alternative services are required to manage non-urgent demand, currently being borne by the ED and the ambulance service, particularly in out of hours.

## Introduction

The pressures of patient demand on emergency care services continue to be reported in England evidenced by declining performance of Emergency Departments (EDs) against the national four hour performance target [1] and increased crowding, [2] evidenced by higher numbers of 12 hour trolley waits and diversions of ambulances [3, 4]. The ambulance service is also under considerable pressure with calls to the ambulance service doubling in the last 10 years (reaching over 9 million calls) [5].

It has been reported for some time that a proportion of attendances to EDs are amenable to management in settings providing a lower level of care such as primary care, walk-in centres and urgent care centres [6, 7]. These attendances (variously described as non-urgent, avoidable or inappropriate) [8] are an indicator of emergency and urgent care systems that could perform better, particularly if such attendances are conveyed by ambulance [9]. In part due to the differences in definitions, the precise size and nature of the problem of non-urgent attendances remains unclear, with some estimates of attendances ranging from 4 to 40% [8, 10]. However, essentially these patients are those not requiring the full range of facilities offered by a typical consultant led Emergency Department in order to manage their healthcare problem.

Good quality data is required to accurately estimate the numbers and types of patients who might be managed in more appropriate settings and reduce pressure on emergency care services. We conducted a retrospective longitudinal analysis of routinely available data to:

1. Define a proportion of non-urgent ED attendances that were amenable to management in alternative non-emergency settings such as GP or nurse led urgent care facilities in either hospital or community settings.
2. Measure and profile the extent of these attendances across Yorkshire and Humber as a whole between April 1^st^ 2011 to March 31^st^ 2014.

## Methods

The National Health Service (NHS) Health Research Authority (HRA): National Research Ethics Service Committee South West Exeter provided ethical approval for this study (Research Ethic Committee Reference: 14/SW/1014). As this study involved the analysis of pseudonymised routinely collected patient data it was deemed suitable for proportionate review. All adult (>16 years) attendances to type I EDs (consultant-led, multi-specialty 24-hour services with full resuscitation facilities and designated accommodation for the reception of ED patients) across the Yorkshire and Humber region of England were assessed by analysing three years of complete Hospital Episode Statistics Accident and Emergency (HES A&E) data for all 13 acute trusts in Yorkshire and Humber from April 1^st^ 2011 to March 31^st^ 2014.

### Data collection

We obtained patient level pseudonymised data on the following: *Age*, *sex*, *date of attendance*, *attendance category (first or follow up attendance)*, *incident location (home*, *public place*, *work or educational establishment)*, *arrival mode (ambulance or other)*, *source of referral (whether self-referred or referred by a professional in another organization)*, *attendance*, *disposal (including whether discharged*, *admitted or referred for follow up)*, *time of arrival*, *time to assessment*, *time to treatment and time to departure*, *department type (type 1*, *2 or 3 ED)*, *location of incident*, *clinical investigations*, *clinical treatments and diagnosis*.

### Definition of non-urgent attendance

A validated process based definition of non-urgent attendance previously published by one of the study authors was adapted for this study [6]. As with this original definition, our approach applied the definition to patients who attended a type 1 ED, as a first attendance, but who were subsequently identified as not receiving investigations, treatments or referral that required the facilities of a type 1 ED. For the purposes of this definition all follow up attendances, whether planned or unplanned were considered urgent. Attendances at single specialty emergency departments, or centres designed for non-emergency patients were not included in the analysis.

Following consultation with our expert steering group (including clinicians, NHS managers and patient representatives) lists of investigations and treatments that did not require the facilities of a fully staffed ED and that could routinely be provided in a non-emergency care setting, such as primary care, were identified. The treatment, investigation and disposal HES codes taken as indicating non-urgent ED attendance are shown in Table 1 below.

**Table 1:** List of investigations and treatments identifying non-urgent ED attendances with corresponding HES A&E codes.

**Code**	**Investigation**
24 or blank	None
06	Urinalysis
21	Pregnancy test
22	Dental investigation
**Code**	**Treatment**
07	Prescriptions
22	Guidance/advice only
30	Recording vital signs
56	Dental treatment
57	Prescription
99 or blank	None
**Code**	**Disposal**
02	Discharged—following treatment to be provided by GP
03	Discharged—did not require any follow-up treatment
12	Left department before being treated

Our definition of non-urgent attendance was therefore developed as follows:

1. Not investigated in ED (except by urinalysis, pregnancy test or dental investigation)
2. Not treated in ED (except by prescription, recording vital signs, dental treatment or guidance/advice)
3. Discharged completely from care in ED or referred to their GP

This could be summarised as identifying “first attendance with some recorded treatments or investigations all of which may have reasonably been provided in a non-emergency care setting, followed by discharge home or to GP care.”

### Identifying non-urgent attendances

As this analysis was limited to data on first time attendances at type 1 EDs, in the first instance all attendances at type 1 EDs (excluding type 2 and type 3) were identified using the appropriate code within ‘department type’ field. First time attendances were then identified from this subset using the appropriate code within the ‘attendance category’ field. This subset of attendances was used as the dataset for this analysis and those attendances which met the criteria of our process based definition were selected by identifying the corresponding HES codes detailed in Table 1. In addition a proportion of the treatment and investigation fields in HES A&E episodes are blank and it is not clear whether this denotes that no investigation or treatment took place or if the data is missing. If a treatment or investigation field was blank, but at least one other treatment or investigation code was completed, then this was interpreted as no treatment or investigation. However, where all the treatment and investigation variables have blank codes, non-urgent attendance is considered not known or ‘missing’.

Patients who left before being seen have also been coded as non-urgent attenders. Irrespective of the presence or absence of treatment and investigation codes, patients who were referred to the ED or fracture clinics, were admitted, died in the Emergency Department, or left the Emergency Department having refused treatment were all classified as urgent attenders.

### Statistical analysis

Time series regression models were used to analyse trends in non-urgent attendance over the three year period allowing for autocorrelation and adjusting for seasonality using Fourier terms. Using summary statistics non-urgent attenders were examined by variables hypothesised to influence them as follows: Age at arrival, time of day and day of week and mode of arrival. For the purposes of our study when analysing the impact of time of day we defined an out of hours period and an in hours period as follows: 8am to 6pm Monday-Friday and all weekend was out of hours. Odds ratios were calculated to compare non-urgent attenders between groups. The distribution of waiting, treatment and total time spent in the department were compared between urgent and non-urgent attendances using non-parametric Mann-Whitney U tests. Analysis was performed using SPSS (version 22.0, IBM, Chicago, Illinois) and the statistical computing package R (www.R-project.org).

## Results

### Attendance types

**Fig 1:**
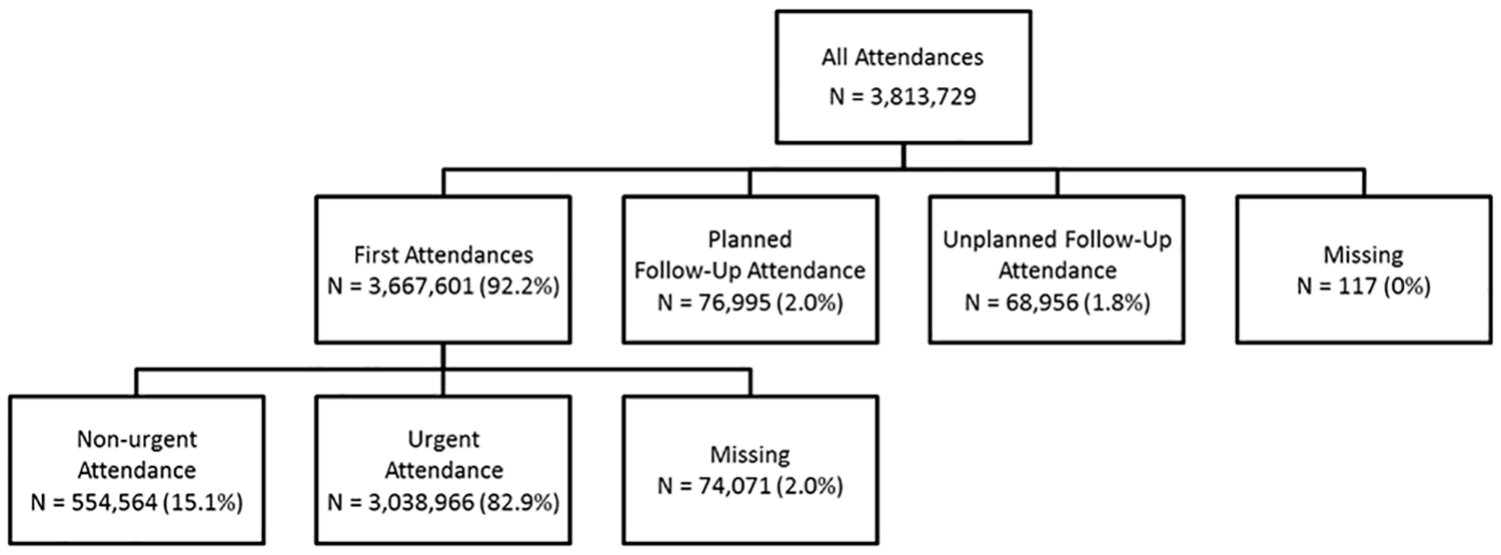
Classification of all attendances.

Fig 1 shows the classification of attendance type by the application of our process for identifying non-urgent attendances to ED. There were a total of 3,813,729 ED attendances across 13 acute trusts between April 1^st^ 2011 and March 31^st^ 2014, of which 3,667,601 (96.2%) were identified as first time ED attendances. 554,564 (15.1%) cases met our definition of non-urgent attendance, whose care could routinely be provided by alternative care services. Overall, non-urgent attendances decreased significantly over the three years, equivalent to 204.7 attendances per year (95% CI: -294.4 to -115.0), P<0.001, although this was clinically equivalent to less than one patient per day across the region.

### Non-urgent attendances

#### Age at arrival

**Fig 2:**
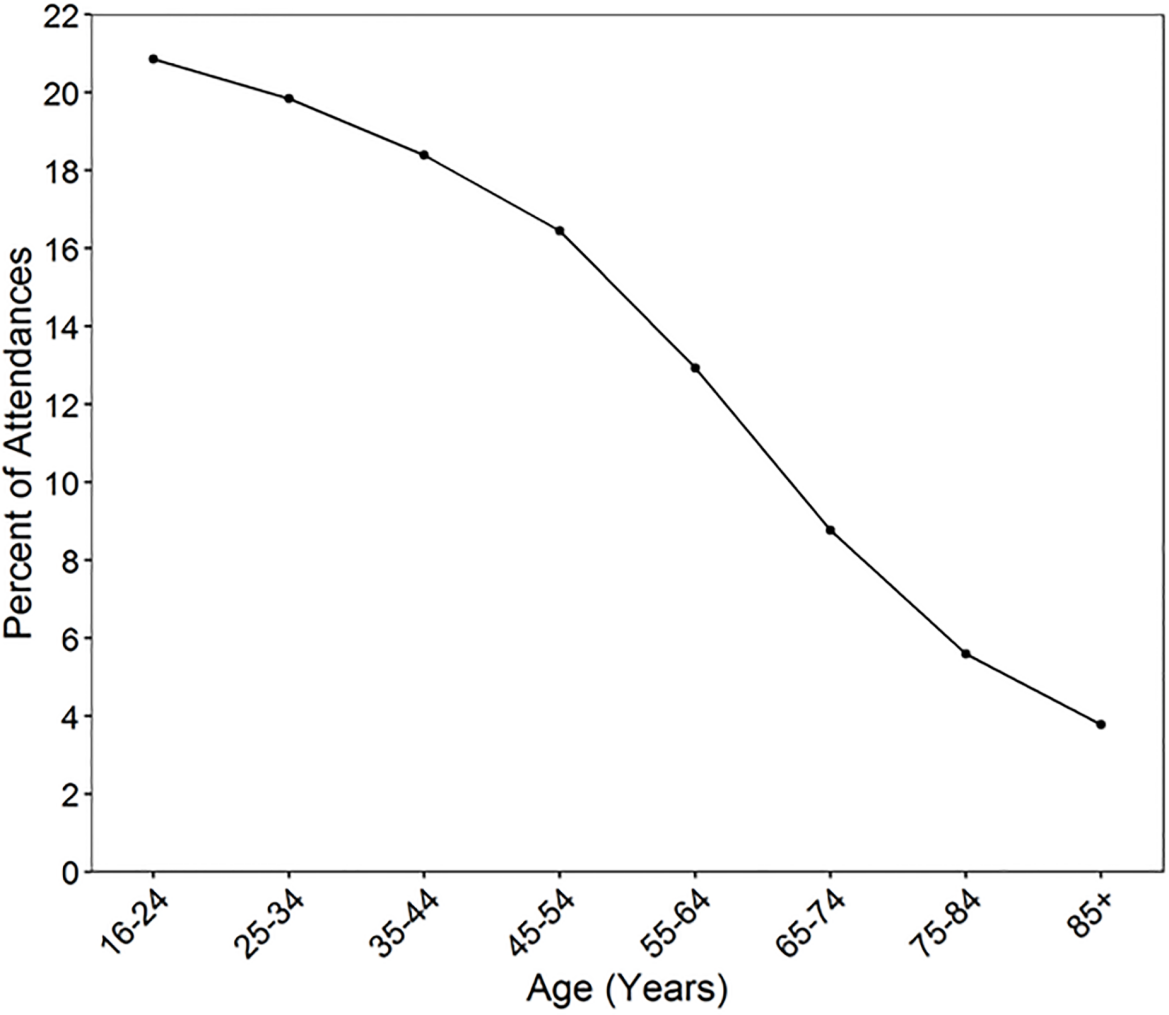
% of non-urgent attendances by age (categories).

Examining attendance type by age category, the highest proportion of non-urgent attenders were in the youngest age group (16–24) and the lowest in the oldest age group (85+) (See Table 2). 365,716 of 554,564 (65.9%) non-urgent attenders were under 45 years of age. The relationship between age categories and percentage of non-urgent attendances is also illustrated in Fig 2, with a change in the slope of the graph apparent at two points (at the 45–54 and the 65–74 age groups). As a result, we categorised age into three age groupings (16–44, 45–64 and 65+) to compare the odds of a non-urgent attendance between them. The odds of a non-urgent attendance were significantly higher for the 16–44’s compared to the 45–64’s (odds ratio: 1.42, 95% CI: 1.41 to 1.43, P<0.001) and compared to the 65+’s (odds ratio: 3.81, 95% CI: 3.78 to 3.85, P<0.001).

**Table 2:** Total attendances, non-urgent and urgent attendances by age.

Age group	Non-urgent attendances N (%)	Urgent attendances N (%)	Missing N (%)	Total N (%)
16–24	144,724 (20.9)	530,472 (76.5)	18,551 (2.7)	693,747 (100.0)
25–34	124,374 (19.8)	483,968 (77.2)	18,245 (2.9)	626,587 (100.0)
35–44	96,618 (18.4)	415,197 (79.1)	13,377 (2.5)	525,192 (100.0)
45–54	81,092 (16.5)	401,511 (81.5)	10,234 (2.1)	492,837 (100.0)
55–64	47,941 (12.9)	316,608 (85.4)	6,077 (1.6)	370,626 (100.0)
65–74	29,836 (8.8)	306,931 (90.2)	3,690 (1.1)	340,457 (100.0)
75–84	20,366 (5.6)	341,502 (93.7)	2,585 (0.7)	364,453 (100.0)
85+	9,613 (3.8)	242,777 (95.7)	1,312 (0.5)	253,702 (100.0)
Total	554,564 (15.1)	3,038,966 (82.9)	74,071 (2.0)	3,667,601 (100.0)

#### Mode of arrival

The proportion of non-urgent attenders varied by arrival mode (see Table 3), with the odds of a non-urgent attendance arriving by ambulance significantly lower than by other methods (odds ratio: 0.395, 95% CI: 0.392 to 0.398, P<0.001). Over the three years there was a decrease of -0.82% per year (95% CI: -0.95 to -0.68, P<0.001) in the proportion of attendances arriving by ambulance defined as non-urgent. This is a decrease from approximately 9.8% in April 2011 to 7.3% in March 2014.

**Table 3:** Attendance type by mode of arrival.

	Non-urgent N (%)	Urgent N (%)	Missing N (%)	Total N (%)
Ambulance	110,605 (8.5)	1,175,428 (90.6)	11,868 (0.9)	1,297,904 (100.0)
Other	443,924 (18.7)	1,863,435 (78.6)	62,203 (2.6)	2,369,559 (100.0)
Not Known	35 (25.4)	103 (74.6)	0 (0.0)	138 (100.0)
Total	554,564 (15.1)	3,038,966 (82.9)	74,071 (2.0)	3,667,601 (100.0)

#### Time of arrival

**Fig 3:**
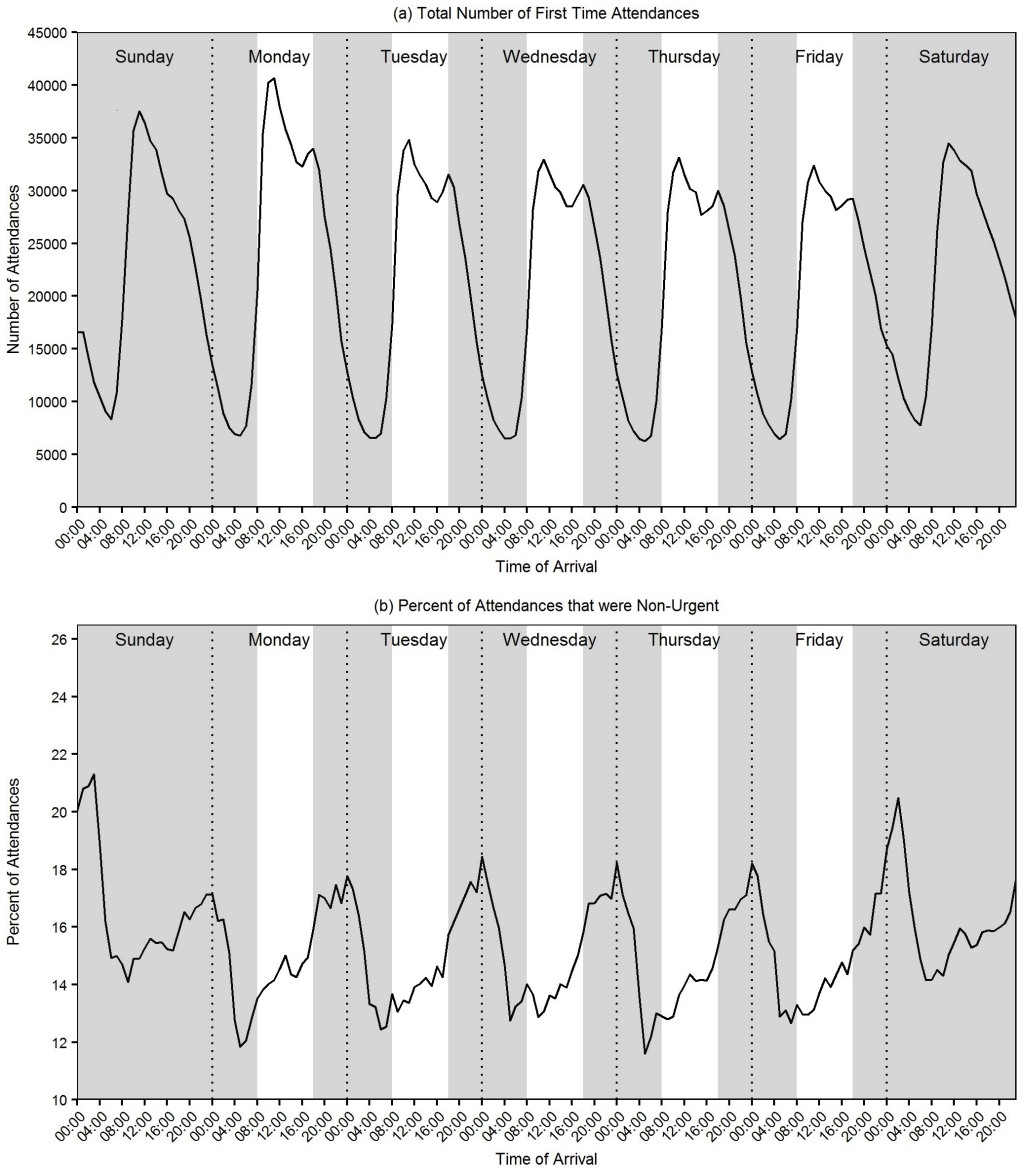
Proportion of first time attendances and non-urgent by day of week and time of day.

Of the total number of non-urgent attendances, almost two-thirds presented in the out of hours period (346,274/554,564; 62.4%). The odds of a non-urgent attendance are significantly greater for patients attending in the out of hours versus in hours (odds ratio: 1.19, 95% CI: 1.18 to 1.20, P<0.001). The average pattern of both all first time attendances and non-urgent attendances by day of week and time of day is shown in Fig 3. The pattern of all first time attendances showed the greatest numbers of attendances in the in hours period, with peaks in attendance around midday across all days of the week (Fig 3a). However, the proportion of attendances that were non-urgent peaked in the very late night / early morning out of hours period across all days of the week, with the highest peaks at the weekend, on a Sunday between 03:00 and 04:00 (2513/11862; 21.2%) (Fig 3b). Non-urgent attendances declined to their lowest point in the early hours of the morning, around 6am, with the lowest average rate on Thursday between 5am and 6am (727/6277; 11.6%) (Fig 3b).

#### Age and time period of arrival (in hours and out of hours)

Across all age groups the majority of non-urgent attenders presented in the out of hours period, and over two thirds (235,637/346,273; 68.0%) of non-urgent presentations in patients aged 16–44 arrived out of hours. The odds of a non-urgent attendance out of hours were significantly higher for the 16–44’s when compared to the two older age groupings, 45–64’s (OR = 1.24, 95% CI: 1.22 to 1.25, P<0.001) and 65+’s (OR = 1.38, 95% CI: 1.35 to 1.40, P<0.001).

**Fig 4:**
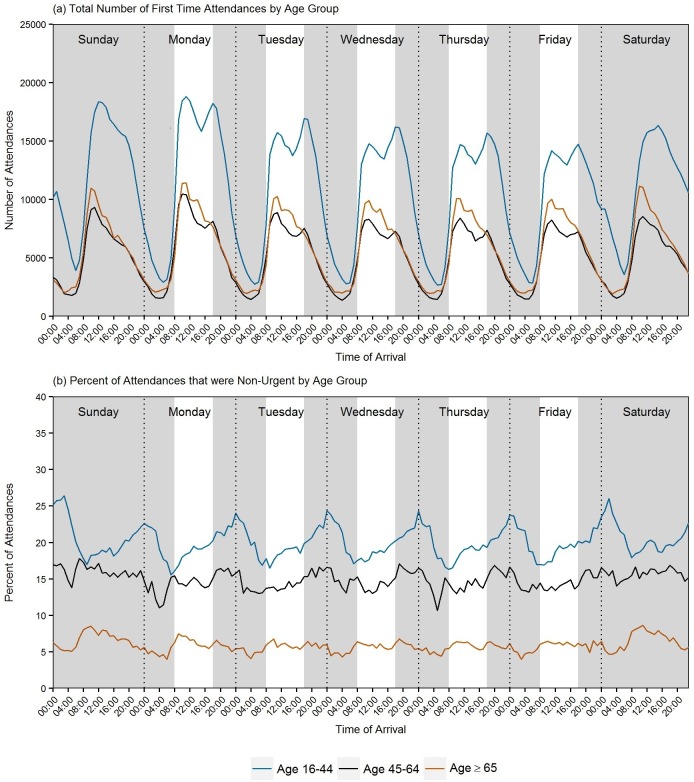
Pattern of first time attendances and non-urgent by day of week/time of day and age.

Fig 4 shows the distribution of all first time attendances and non-urgent attendances by time of day and day of week when our sample was split by age into the three age groupings. The distribution of total first time attendances were similar in the 45–64 and 65+ age categories (Fig 4a) and followed the pattern for all first time attendances described above in Fig 3a, with peaks in attendance around midday across all days of the week. The distribution of first time attendances in the 16–44’s differed to the two older age categories between Monday and Friday, with peaks in attendances around 18.00 hours. The % of attendances that were non-urgent and the distribution of these attendances by time of day /day of week were different in the three age groupings. A greater proportion of attendances were non-urgent across all the days of the week and times of the day for those 16–44 compared to the 45–64 and 65+ age categories (Fig 4b). It was also in this youngest age grouping that the peaks in non-urgent were distributed in the very late night /early morning across all days of the week, with a peak on a Sunday morning 03:00–04:00 (2,101/7,966; 26.4%).

#### Arrival mode by age group

64,958/365716 (17.8%) of non-urgent attendances in the 16–44’s arrived by ambulance compared to 26530/129033 (24.2%) in the 45–64’s and 19120/59815 (32.0%) in the 65+ age category. The odds of a non-urgent attendance being conveyed by ambulance were significantly lower for the 16–44’s when compared with the older two age groupings, 45–64’s (OR = 0.83, 95% CI: 0.82 to 0.85, P<0.001) and 65+’s (OR = 0.46, 95% CI: 0.45 to 0.47, P<0.001).

#### Arrival mode and time period of arrival

A higher proportion of the non-urgent attendances that were conveyed by ambulance were in the out of hours period compared with the in hours period (Table 4), with 24.1% of non-urgent conveyed by ambulance out of hours versus 13.0% in hours OR = 2.12, 95% CI: 2.09 to 2.15, P<0.001). Of the total non-urgent conveyed by ambulance, 75.5% were conveyed in the out of hours period. Our time series model shows that the percent of non-urgent attendances arriving by ambulance out of hours decreased by -0.4% per year (95% CI: -0.7 to -0.1, P = 0.014). This is a decrease from approximately 24.7% in April 2011 to 23.5% in March 2014.

**Table 4:** Non-urgent attendances by arrival mode and time period of arrival.

	In Hours N (%)	Out of Hours N (%)	Total N (%)
**Ambulance**	27,151 (13.0)	83,457 (24.1)	110,608 (19.9)
**Other**	181,129 (87.0)	262,792 (75.9)	443,921 (80.0)
**Not Known**	11 (0.0)	24 (0.0)	35 (0.0)
**Total**	208,291 (100.0)	346,273 (100.0)	554,564 (100.0)

#### Non-urgent attendance via ambulance by age and time period of arrival

**Fig 5:**
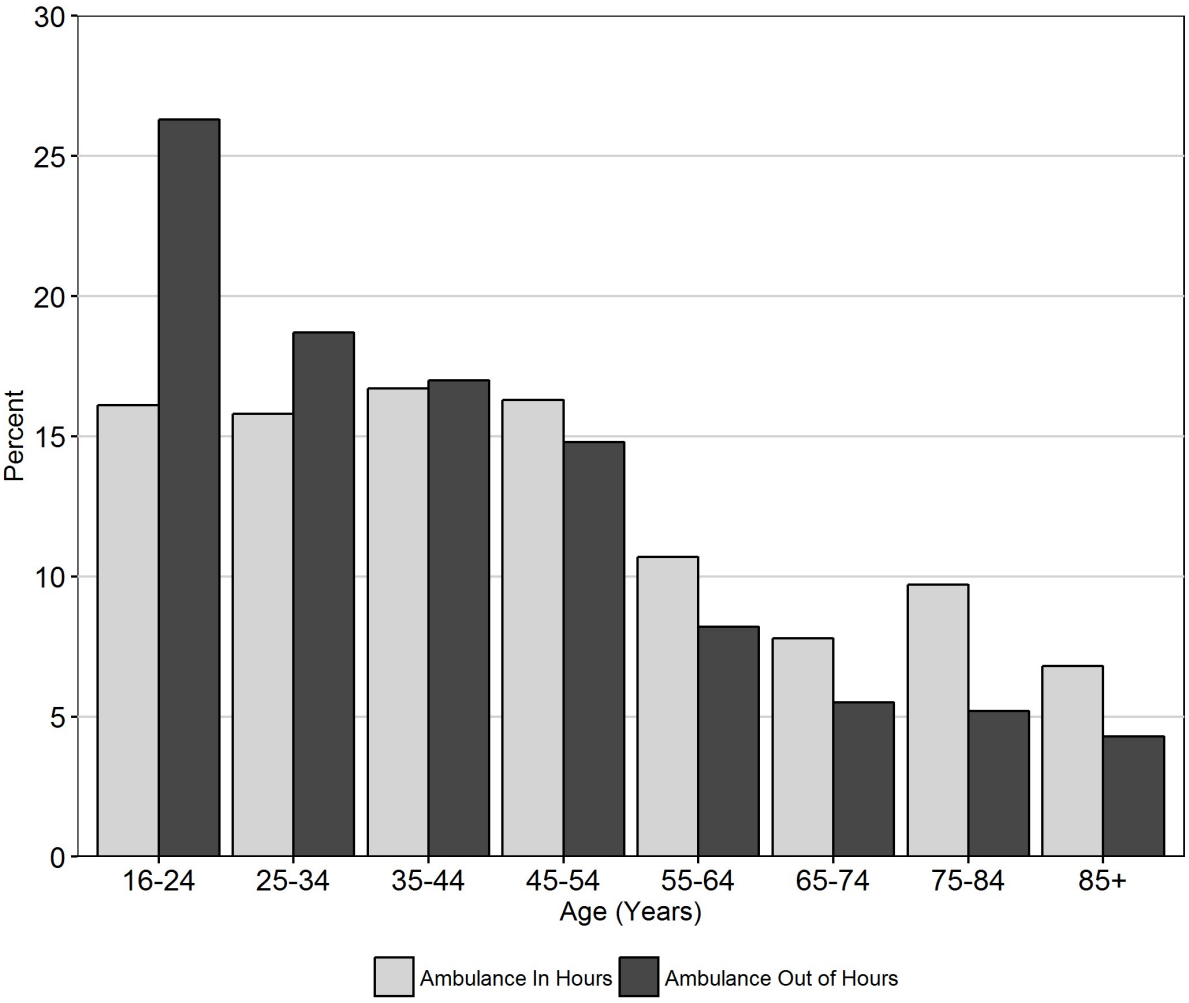
% of non-urgent attendances presenting by ambulance by age group and time period.

Examining those patients who arrived by ambulance we found that that during the in hours period patients in the youngest three age categories (<45 years) made up 48% of the total of non-urgent attendances by ambulance, rising to 62% in the out of hours period (Fig 5).

### ED performance indicators by attendance type (urgent or non-urgent)

#### Waiting times, treatment times and total department times

The impact of non-urgent attenders on average departmental performance is detailed in Table 5 below. The treatment time and total time in department were significantly less for non-urgent versus urgent attendances (Using a non-parametric Mann-Whitney U test all differences are statistically significant P<0.001)

**Table 5:** ED waiting times, treatment times and total department times by attendance type (urgent or non-urgent).

	Non-urgent			Urgent		
	N	Mean (SD)	Median (IQR)	N	Mean (SD)	Median (IQR)
**Waiting Time**	432,141	72.9 (55.4)	61 (29–106)	2,708,436	70.0 (55.7)	57 (27–102)
**Treatment Time**	432,141	36.5 (41.8)	22 (11–46)	2,708,436	94.7 (79.5)	76 (35–136)
**Total Department Time**	432,141	109.4 (63.7)	100 (59–151)	2,708,436	164.7 (86.0)	161 (102–219)

## Discussion

### Summary of main results

This retrospective study utilised three years of routine data to understand the patterns and profile of adult non-urgent attendance to EDs in a large English region. The overall rate of non-urgent attendance was 15%, this equates to 3.6 million patient attendances at type I EDs in England in 2016. Non urgent attendances were significantly more likely to occur in the out of hours period compared to in hours, including those non-urgent attendances arriving by ambulance (compared with other means). Patients in the three youngest age categories (under 45 years of age) had significantly greater odds of a non-urgent ED attendance than those in the age categories over 45. They also had significantly greater odds of a non-urgent ED attendance out of hours compared to the age categories over 45. Around 20% of the total number of non-urgent attendances arrived by ambulance, with non-urgent attendances by ambulance (compared with other means) more than twice as likely out of hours as in hours. Once in the ED, we found that both time to treatment and total time in ED were significantly less for non-urgent attendances versus urgent attendances.

### Comparability of findings

Previous studies have reported a wide variation in the rates of attendances that might be managed in alternative settings to the ED, such as primary care [8,11–17]. For example, a systematic review of the evidence [8] found rates ranging from 4% to 90% in inappropriate use of emergency services. The range in the reported proportions of ED attendances identified in the literature as being eligible for care outside of ED is in part due to the absence of an agreed definition for such attendances. Studies examining this issue were also carried out in different settings and using different methods and data sources to identify cases. For example some studies have identified patients presenting with low acuity presentations based on triage scores in defined patients (such as self-referred/non ambulance patients) [11, 12] while others have retrospectively reviewed ED case notes [13, 14]. Many studies were based in a single centre and on a small sample of patients [14, 16]. Our finding of 15% is lower than some UK study estimates [15, 16] although our study definition did not limit the identification of non-urgent attendances to non-ambulance, self-referred presentations as previous studies have done. A number of studies have identified a similar relationship to our study between younger age and increases rates of attendances suitable for care in other settings [8, 11, 13, 15]. We found a greater number of attendances were non-urgent in the out of hours period compared to the in hours period and a previous study also reported the highest peak in ‘inappropriate’ attendances in the evening between 20.00 and 22.00 hours [16] and increased odds of ‘inappropriate’ attendance at weekends and bank holidays [17].

### Strengths and weaknesses of study

A key strength of this study is using one large region of the UK. The region represents large urban, suburban and rural areas along with a range of Emergency Departments including Major Trauma Centres and smaller type I departments, which we believe is representative of the national picture. Previous work in this area was carried out in either a single ED or a small number of EDs and a strength of this study is applying an objective definition of non-urgent attendance across 13 NHS trusts, some of which had more than one Type 1 ED. We also included patients transported to the ED by ambulance, rather than limiting the analysis to self-referred patients. If we had undertaken the latter approach we would have found an even higher rate of non-urgent attendances, but not identified an important sub-set of non-urgent attendances that are also using other resources in the NHS inappropriately (in this case the ambulance service) and are therefore amenable to intervention. The application of a previously validated, objective, reproducible methodology for identifying non-urgent attendances which can be retrospectively applied to large routine datasets is a further strength of this study. The relative advantage of our definition of non-urgent attendance compared to others in the literature is that it is based on the processes of care that the patient received in the ED. Alternative approaches include the use of triage scores, which is based only on an initial assessment of urgency and therefore does not necessarily take into account important factors such investigations and treatments subsequently undertaken. Using recorded diagnoses [5] to identify cases eligible for alterative care to ED does not take into account the fact that investigations only available in ED may be required to rule out a more serious complaint than the one eventually recorded. Our definition assumed that all investigations and treatments received by study patients were clinically appropriate. This assumption may underestimate the true rate of non-urgent attendance, as there may be a tendency for over use of ED investigations and treatments (‘defensive medicine’) in some instances which was not measured here.

There are weaknesses in the analysis of routine datasets such as those utilised in this study, with potential bias introduced by missing data and a lack of information about the methods used to code the data. The data did not code the clinical case mix of attendances in a way that could identify important sub groups for intervention. The application of a retrospective definition of non-urgent attendance also does not capture the range of reasons related to the structure of health services (such as availability or access to alternative services) or to patient factors such as perceived seriousness of their presentation.

### Clinical implications

Our study has demonstrated that the number of non-urgent attendance at type I EDs represents a significant proportion of the clinical workload of departments. This workload is significantly more likely to apply to younger people rather than older people. These attendances may reflect problems patients experience in accessing care in alternative settings, such that they have no alternative but to attend the ED. They may also represent confusion about where to access care most appropriately and also represent the desire (particularly in younger people) for convenient and accessible care at a time that suits them. This study demonstrates that patients attending ED non-urgently spent significantly less time in departments (median time of just over an hour and a half) than urgent attendances, which may act as an incentive for patients to use the ED. These attendances could be managed in alternative urgent care settings which would have a twofold benefit for both the service and patients. Diverting these types of attendances to alternative settings may have a considerable ‘decongesting’ effect on crowded departments, allowing scarce ED resources to be concentrated on those patients in greatest need of the facilities and care provided by a major (type I) emergency department. In addition, patients presenting non-urgently to ED are likely to benefit more clinically from care in an alternative setting where continuity of care may be more appropriate for their type of problem. Systems need to be configured in such a way that patients receive the care they require in a convenient manner but without accessing higher levels of care than actually needed. However such reconfiguration needs to take into account the way patients (especially younger age groups) access care and the time of day that it is more likely to be needed. In addition, there are risks that providing comprehensive alternatives would lead to provider induced demand higher than anticipated.

The rate of non-urgent attendances by ambulance is particularly concerning as the findings from our study indicate potential misuse of the ambulance service. There is an urgent need to examine alternatives to conveyance to hospital for sub-groups of ambulance patients including younger age groups, late at night, where alcohol may potentially be involved in the presentation. The challenge for services is to identify the sub-groups of patients who would benefit most from a safe alternative to transportation to hospital whilst ensuring the most appropriate responses are in place to provide more appropriate care.

### Further research

Further research is required to identify specific clinical groups who would most benefit from an alternative approach to care from a consultant led type I ED, particularly those who are transported non-urgently via ambulance. Interventions such as alternative service models need to be carefully evaluated to ensure they provide a safe and cost effective alternative to current provision. Other factors hypothesised to impact on use of ED for non-urgent conditions such as proximity of patient dwelling to ED merit further work. Studies identifying potential variation in ED rates of non-urgent attendance and associated service provision are also required.

### Conclusion

Overall, younger people are significantly more likely as older people to use the ED to obtain healthcare that could be provided in a primary care type setting and more likely than older people to do this in out of hours. This may reflect changing attitudes and patterns of use of emergency services in this patient group. Overall patient use of ambulances for non-urgent attendances to ED in out of hours is notable.

## References

[1] Monitor. *A&E Delays*: *why did patients wait longer last winter*? London: Monitor; 9 2015.

[2] Blunt I. Quality Watch. Focus on: A&E attendances: Why are people waiting longer? London: A joint report from The Health Foundation and Nuffield Trust; 2014.

[3] Boyle A, Higginson I, Smith S, Henderson K. *Crowding in the Emergency Department*. 3^rd^ ed: College of Emergency Medicine; 2014.

[4] Appleby J and Dayan M. Nuffield Winter Insight: Briefing 3, The Ambulance Service. Nuffield Trust, April 2017.

[5] Health and Social Care Information Centre (HSCIC), Ambulance Services 2014–15. June 2015. http://www.hscic.gov.uk/catalogue/PUB17722/ambu-serv-eng-2014-2015-rep.pdf]

[6] Lowy A, Kohler B, Nicholl J. Attendance at accident and emergency departments: Avoidable or inappropriate? *J Pub Health Med* 1994;16:134–140. 7946485 10.1093/oxfordjournals.pubmed.a042947

[7] Dale J, Green J, Reid F, Glucksman E. Primary care in the accident and emergency department: I. Prospective identification of patients. *BMJ* 1995;311(7002):423–6. 7640591 10.1136/bmj.311.7002.423 PMC2550493

[8] Carret ML, Fassa AC, Domingues MR. Inappropriate use of emergency services: a systematic review of prevalence and associated factors. *Cad Saude Publica*. 2009;25(1):7–28. 19180283 10.1590/s0102-311x2009000100002

[9] MCRU Report Nicholl J, Coleman P, Jenkins J, et al. The emergency and urgent care system. Final report to Department of Health 2006–2010. Sheffield: Medical Care Research Unit.

[10] Bickerton J, Davies J, Davies H, Proctor S, Apau D. Streaming primary urgent care: a prospective approach. *Primary Health Care Research Dev* 2012:13;142–152. 10.1017/S146342361100017X 21774867

[11] Afilalo J, Marinovich A, Afilalo M, Colacone A, Léger R, Unger B, et al Nonurgent emergency department patient characteristics and barriers to primary care. *Acad Emerg Med* 2004;11(12):1302–10. 15576521 10.1197/j.aem.2004.08.032

[12] Nagree Y, Ercleve TN, Sprivulis PC. After hours general practice clinics are unlikely to reduce low acuity patient attendances to metropolitan Perth emergency departments. *Aust Health Rev*: 28 285–91. 15595910 10.1071/ah040285

[13] David M, Schwartau I, Anand Pant H, Borde T. Emergency outpatient services in the city of Berlin: Factors for appropriate use and predictors for hospital admission. *Eur J Emerg Med* 2006;13(6):352–7. 17091058 10.1097/01.mej.0000228451.15103.89

[14] Thompson MI, Lasserson D, McCann L, Thompson M, Heneghan C. Suitability of emergency department attenders to be assessed in primary care: survey of general practitioner agreement in a random sample of triage records analysed in a service evaluation project. *BMJ Open* 2013;3(12):e003612 doi: 10.1136/bmjopen-2013-003612 24319279 10.1136/bmjopen-2013-003612 PMC3855530

[15] Cowling TE, Cecil EV, Soljak MA, Lee JT, Millett C, Majeed A, et al Access to primary care and visits to emergency departments in England: a cross-sectional, population-based study. *PLoS One* 2013;8(6):e66699 doi: 10.1371/journal.pone.0066699 23776694 10.1371/journal.pone.0066699 PMC3680424

[16] Selasawati HG, Naing L, Wan Aasim WA, Winn T, Rusli BN. Inappropriate utilization of emergency department services in Universiti Sains Malaysia hospital. *Med J Malaysia* 2004;59(1):26–33. 15535332

[17] McHale P, Wood S, Hughes K, Bellis MA, Demnitz U, Wyke S. Who uses emergency departments inappropriately and when—a national cross-sectional study using a monitoring data system. *BMC Med* 2013;11:258 doi: 10.1186/1741-7015-11-258 24330758 10.1186/1741-7015-11-258 PMC3886196

